# The Landscape of the Tumor-Infiltrating Immune Cell and Prognostic Nomogram in Colorectal Cancer

**DOI:** 10.3389/fgene.2022.891270

**Published:** 2022-05-12

**Authors:** Jiateng Zhong, Yu Qin, Pei Yu, Weiyue Xia, Baoru Gu, Xinlai Qian, Yuhan Hu, Wei Su, Zheying Zhang

**Affiliations:** ^1^ Department of Pathology, The First Affiliated Hospital of Xinxiang Medical University, Xinxiang, China; ^2^ Department of Pathology, Xinxiang Medical University, Xinxiang, China; ^3^ Department of Gynecology, The Fourth Clinical College of Xinxiang Medical University, Xinxiang, China; ^4^ Xinxiang Key Laboratory of Tumor Vaccine and Immunotherapy, Institute of Precision Medicine, Xinxiang Medical University, Xinxiang, China

**Keywords:** CIBERSORT, immunotherapy, colorectal cancer, prognosis, nomogram

## Abstract

Tumor-infiltrating immune cells are associated with prognosis and immunotherapy targets in colorectal cancer (CRC). The recently developed CIBERSORT method allows immune cell analysis by deconvolution of high-throughput data onto gene expression. In this study, we analyzed the relative proportions of immune cells in GEO (94 samples) and TCGA (522 samples) CRC data based on the CIBERSORT method. A total of 22 types of tumor-infiltrating immune cells were evaluated. Combined with GEO and TCGA data, it was found that naive B cells, M2 macrophages, and resting mast cells were highly expressed in normal tissues, while M0 macrophages, M1 macrophages, activated mast cells, and neutrophils were highly expressed in tumors. Moreover, we constructed a prognostic model by infiltrating immune cells that showed high specificity and sensitivity in both the training (AUC of 5-year survival = 0.699) and validation (AUC of 5-year survival = 0.844) sets. This provides another basis for clinical prognosis. The results of multiple immunofluorescence detection showed that there were differences in the results of bioinformatics analysis. Neutrophils were highly expressed in normal tissues, and M2 macrophages were highly expressed in tumor tissues. Collectively, our data suggested that infiltrating immune cells in CRC may be an important determinant of prognosis and immunotherapy.

## Introduction

Colorectal cancer (CRC) is a common malignant tumor of the digestive tract, and its incidence ranks third among the top ten malignant tumors (Bray et al., 2018). Most of the CRC has been found in the late stages, often accompanied by metastasis, which is an important reason for the high mortality rate of CRC patients (Xi and Xu, 2021). The treatment is an important factor affecting the survival rate of advanced CRC. Clinical treatment of advanced CRC mainly includes chemotherapy, radiotherapy, and traditional Chinese medicine treatment. Chemotherapy is the main treatment for advanced CRC, which can reduce cancer to a certain extent, but it also increases the toxicity and side effects (Sun et al., 2021). There is still a lack of targets that can help to select personalized treatment options.

The progress of tumors is related to not only the growth and dissemination of tumor cells but also the infiltrating immune cells. It is the interaction between these different types of cells that promotes the growth of tumors (Binnewies et al., 2018). In the past 20 years, there have been a lot of reports on the correlation between the infiltration of intratumoral immune cells and prognosis in solid tumors (Nirmal et al., 2018; Edlund et al., 2019; Kim et al., 2019). Immunotherapy is a kind of therapy that regulates T-cell activity by co-suppression or co-stimulation. The therapeutic methods of high anti-tumor immune response have shown remarkable clinical effects (Waldman et al., 2020; Zhang and Zhang, 2020). Immunocytes infiltrated in tumors are most likely to be used as drug targets to improve patient survival.

With the development of immunological checkpoint therapy, the distribution of infiltrating immune cells in tumors has become a research topic. The previous studies have mostly used flow cytometry or immunohistochemistry to assess the composition of infiltrating immune cells in tumors (Finotello and Trajanoski, 2018), but these methods are difficult to detect in large quantities. Researchers recently developed the CIBERSORT analysis tool, a new bioinformatics tool, which is a deconvolution algorithm developed by Bindea G (Chen et al., 2018). It can estimate the cell composition of complex tissues based on standardized gene expression data.

Here, we use CIBERSORT to quantify the expression of 22 kinds of immune cells in CRC and normal colon tissues. For the first time, we analyze the relationship between immune cells and survival, tumor size, lymphatic metastasis, and blood vessel metastasis of CRC patients. Also, we establish a prognostic nomogram for predicting survival by immune cells. It is hoped that this study will be helpful for prognosis judgment and immunotherapy in CRC patients.

## Materials and Methods

### Data Collection

The study utilized data from the public databases. Gene expression profile datasets for common CRC patients were obtained from the GEO (Gene Expression Omnibus, https://www.ncbi.nlm.nih.gov/gds), and TCGA databases (The Cancer Genome Atlas, https://portal.gdc.cancer.gov/). The GEO data selected three datasets for the patient to be paired with their normal tissues, namely, GSE32323 (platform GPL750), GSE110223 (platform GPL96), and GSE110224 (platform GPL750), for a total of 94 samples, including 47 normal control samples and 47 tumor tissue samples. The samples of inappropriate values were removed, leaving a total of 67 samples, including 30 normal samples and 37 tumor tissue samples. The TCGA dataset and corresponding clinical information were downloaded from TCGA official website, a total of 522 samples, containing 42 normal control samples and 480 tumor tissue samples. After removing unreasonable data, 245 samples remained which included 13 normal control samples and 232 tumor tissue samples.

After downloading the GEO data, the probe name was annotated, the three-chip data were combined into one, and then the experimental batch correction was performed. Similarly, after downloading TCGA data, the data were sorted into a matrix. The data were then normalized. All the aforementioned steps were completed by R software.

### Assessing the Composition of Immune Cells Infiltrated in Tumors

This study uses CIBERSORT (http://cibersort.stanford.edu/) to assess the relative proportion of 22 invasive immune cell types in each tumor tissue. CIBERSORT is an assessment tool that analyzes the abundance of specific cell types in mixed cells. The analysis can be performed using gene expression profile data. The immune cells infiltrated in tumor tissue can be analyzed for a value for subsequent analysis. These tumor-infiltrating immune cell types include: M1 macrophages, M2 macrophages, M0 macrophages, T follicular helper cells, resting memory CD4 T cells, activated memory CD4 T cells, γδ T cells, CD8 T cells, regulatory T cells, naive CD4 T cells, resting NK cells, activated NK cells, resting mast cells, activated mast cells, memory B cells, resting dendritic cells, activated dendritic cells, naive B cells, monocytes, neutrophils, eosinophils, and plasma cells. For each tumor sample, the sum of the evaluated immune cell type scores is equal to 1. The results with a value of *p* < 0.05 were considered eligible for further analysis. The process is completed by R software.

### Nomograms

A nomogram model was established and validated to predict the prognosis of CRC. Then, the corresponding clinical information from TCGA data was downloaded, and the samples were divided randomly into training and validation (3:1) sets for evaluation and verification. The following clinical information was collected from the database: patient, gender, age, TNM stage, and survival data.

Univariate analysis of TCGA immune cell infiltration results and clinical variables, using *p* < 0.05 variables, naive B cells, activated T cells CD4 memory, T cells follicular helper, M0 macrophages, M1 macrophages, and M2 macrophages established a nomogram of 3-year and 5-year survival rates for patients with CRC.

The method of using the nomogram is as follows: the points on the score of each variable of the patient are matched to the scores on the topmost horizontal line, and then the scores of each variable are added to obtain the total score of each patient, and finally the total score corresponds to the bottom-end 3- and 5-year survival rates.

### Multiplex Immunofluorescence Staining (mIF)

A total of three pairs of colorectal samples and adjacent normal tissues were collected from The First Affiliated Hospital of Xinxiang Medical College. The experiments were performed consistently with the guidelines and regulations of the Committee of Xinxiang Medical University. Informed consent was obtained from all patients. Tissues were fixed and embedded for paraffin section, and the paraffin section was dewaxed and antigen repair and serum sealing were performed; the first primary antibody was added, the corresponding HRP-labeled secondary antibody was added, and CY3 fluorescence enhancer was added. Microwave treatment was followed by the addition of the second primary antibody, the corresponding HRP-labeled secondary antibody, and the FITC fluorescence enhancer. A third primary antibody was added, and the corresponding HRP-labeled secondary antibody and a 647 fluorescence enhancement agent were added. After microwave treatment, the fourth primary antibody was added. DAPI was used to re-stain the nucleus, quenching by spontaneous fluorescence, sealing the slices, and placing the slices under a scanner to collect images. During the final imaging, the nuclei stained with DAPI are blue under the excitation of ultraviolet, and the positive expression is the corresponding fluorescein-labeled red light, pink light, green light, and purple light. All the slides were scanned and observed using a Pannoramic MIDI (3DHISTECH) and CaseViewer C.V.2.4. The antibodies CD19, CD163, C-kit, CD68, NOS2, mast cell tryptase, and CD16 were purchased from Servicebio Technology Co. Ltd. DAPI is used to label the nucleus, CD19 is used to label naive B cells, CD163 is used to label M2 macrophages, C-kit labeled the resting mast cells, CD68 labeled cell M0, NOS2 labeled M1 macrophages, mast cell tryptase labeled activated mast cells, and CD16 labeled neutrophils (Jensen et al., 2014; Yamaguchi et al., 2016; Lv et al., 2017; Sanz et al., 2019; Valent et al., 2019; Lu et al., 2020; Lai et al., 2021).

### Statistical Analysis

The relationship between immune cell type and clinic variables was analyzed by univariate Cox regression. The overall survival was analyzed by Kaplan–Meier curves and assessed using a log-rank test. Nomogram construction was according to multivariate Cox regression and Wilcox test results. The C-index was calculated, and the receiver operating characteristic (ROC) and calibration curve was drawn to evaluate the performance of the nomogram model. The differences in the expression of immune cells between tumor and non-tumor tissue were assessed using an unpaired *t*-test, with *p* < 0.05 being considered statistically significant. All analyses were performed using R version 3.5.1.

## Results

### Analysis of the Immune Cell Infiltration Composition in CRC Tissues Based on the GEO Database

**FIGURE 1:**
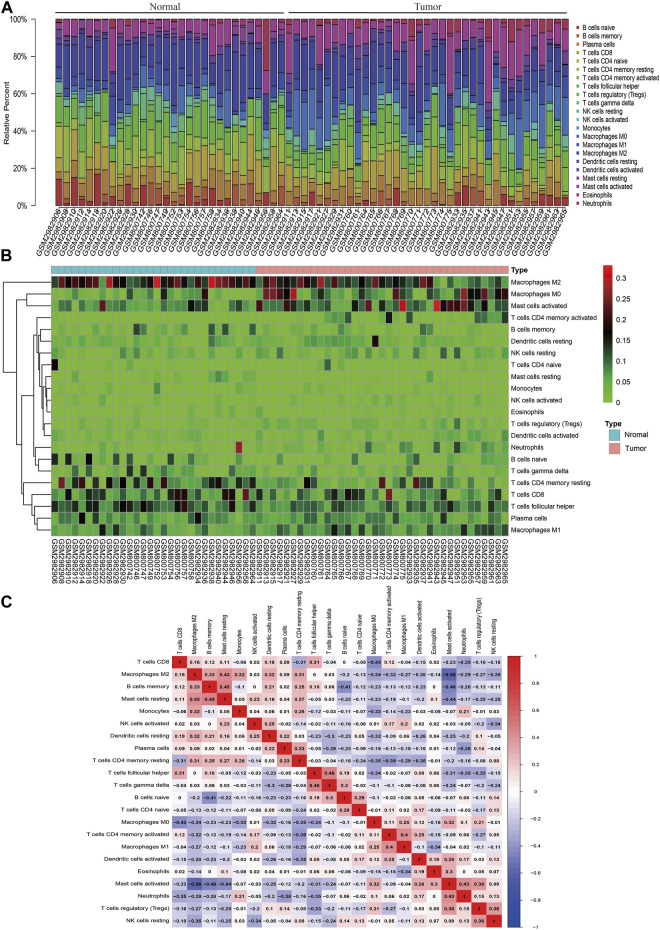
Landscape of immune infiltration in normal and CRC tissues based on GEO data. **(A)** Composition of immune infiltration between paired cancer and normal tissues. The height of the colored column represents the proportion of immune cells. **(B)** Heat map of the 22 immune cell proportions. The genes shown in red are upregulated, and the genes in green are downregulated. The horizontal axis shows the clustering information of samples which were divided into two major clusters. **(C)** Correlation matrix of 22 immune cell expressions. The redder the color, the higher the positive correlation. The bluer the color, the higher the negative correlation.

CIBERSORT algorithm was used to evaluate the distribution of immune cells in tumors and normal tissues of 47 paired CRC patients in the GEO database (Figure 1A). The detailed results are provided in Supplementary Table S1. We can see that there are great differences in the composition of immune cells in different samples, which indicates that the composition of different types of immune cells may be used to diagnose and judge the prognosis of tumors. Hierarchical cluster analysis was used to analyze the difference of immune cell distribution between tumor and normal tissues. We found that there were significant differences in M0 macrophages, M1, M2, and activated mast cells (Figure 1B). It can be seen from the correlation thermogram that there is a significant negative correlation between M2 macrophages and activated mast cells and a strong positive correlation between M0 macrophages (Figure 1C). From Figure 1C, we can find out the correlation between different immune cells, which will play an important role in the analysis of the mechanism in the future.

### Expression of Immune Cells in Cancer and Normal Tissues Based on the GEO Database

**FIGURE 2:**
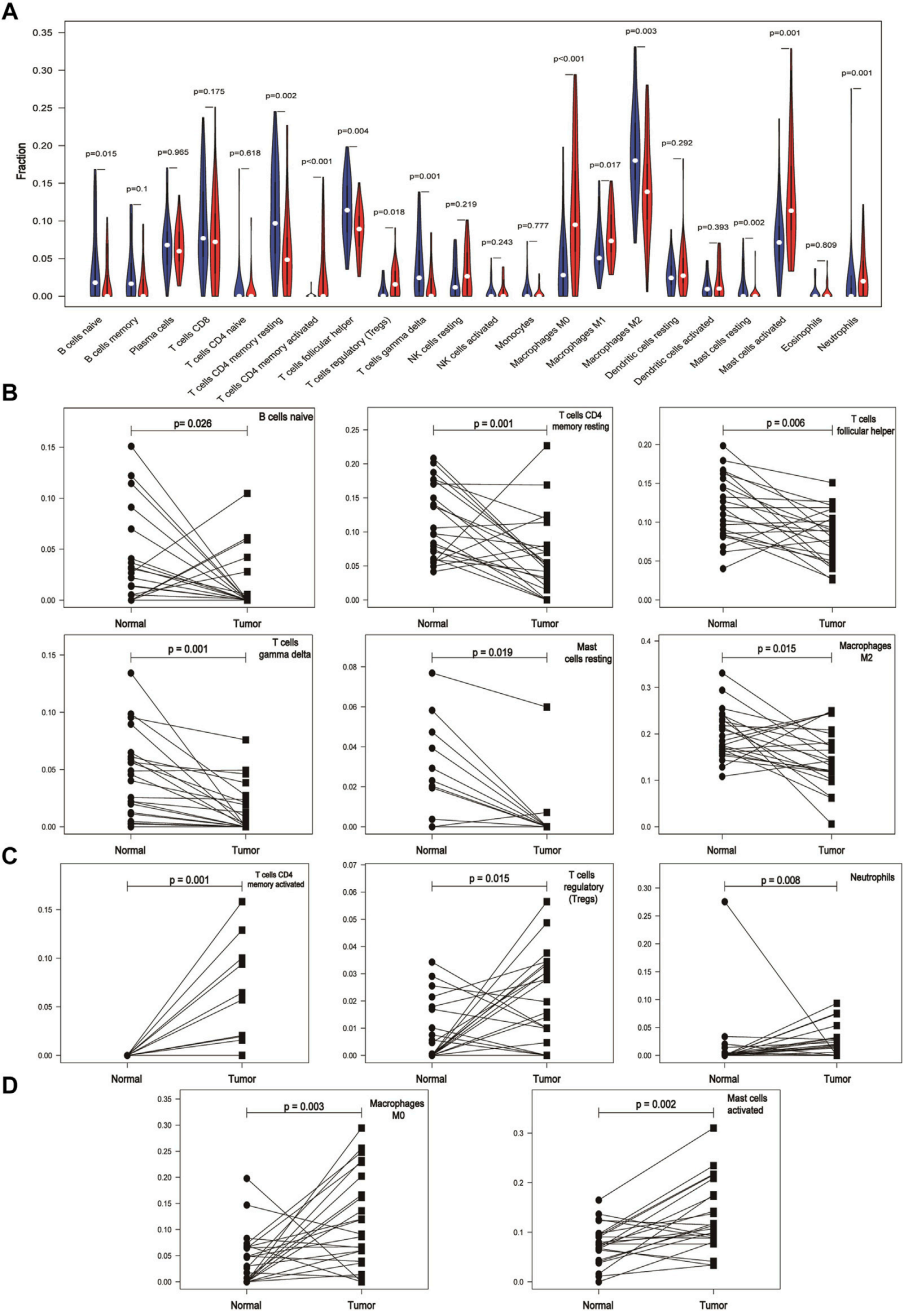
Differential expression of immune cells in cancer and normal tissues based on the GEO database. **(A)** Expression of different immune cells in normal and cancer tissues. The white dots inside the violin indicate the median value. **(B)** Expression of immune cells is lower in cancer tissues than in paired normal tissues. **(C)** Immune cells are mainly expressed in cancer tissues. **(D)** Expression of immune cells is higher in cancer tissues than in paired normal tissues.

By comparing the expression of different types of immune cells in normal and cancer tissues, we found that the expression of naive B cells, resting T cells CD4 memory, activated T cells CD4 memory, T cells follicular helper, regulatory T cells (Tregs), γδ T cells, M0 macrophages, M2 macrophages, resting mast cells, activated mast cells, and neutrophils in cancer and normal tissues was significantly different (*p* < 0.05) (Figure 2A). M1 macrophages were statistically significant in the comparison between the normal and tumor groups of the violin map, but the difference was not statistically significant in the comparison of the paired samples. The fraction of naive B cells, resting T cells CD4 memory, T cells follicular helper, γδ T cells, resting mast cells, and M2 macrophages was higher in normal adjacent tissue than in cancer tissue (Figure 2B). Activated T cells CD4 memory, Tregs, and neutrophils were mainly present in cancer (Figure 2C). M0 macrophages and activated mast cells were increased in cancer compared to normal (Figure 2D). Other immune cells were not significantly altered between tissues.

### The Landscape of Immune Infiltration in CRC Based on TCGA Database

**FIGURE 3:**
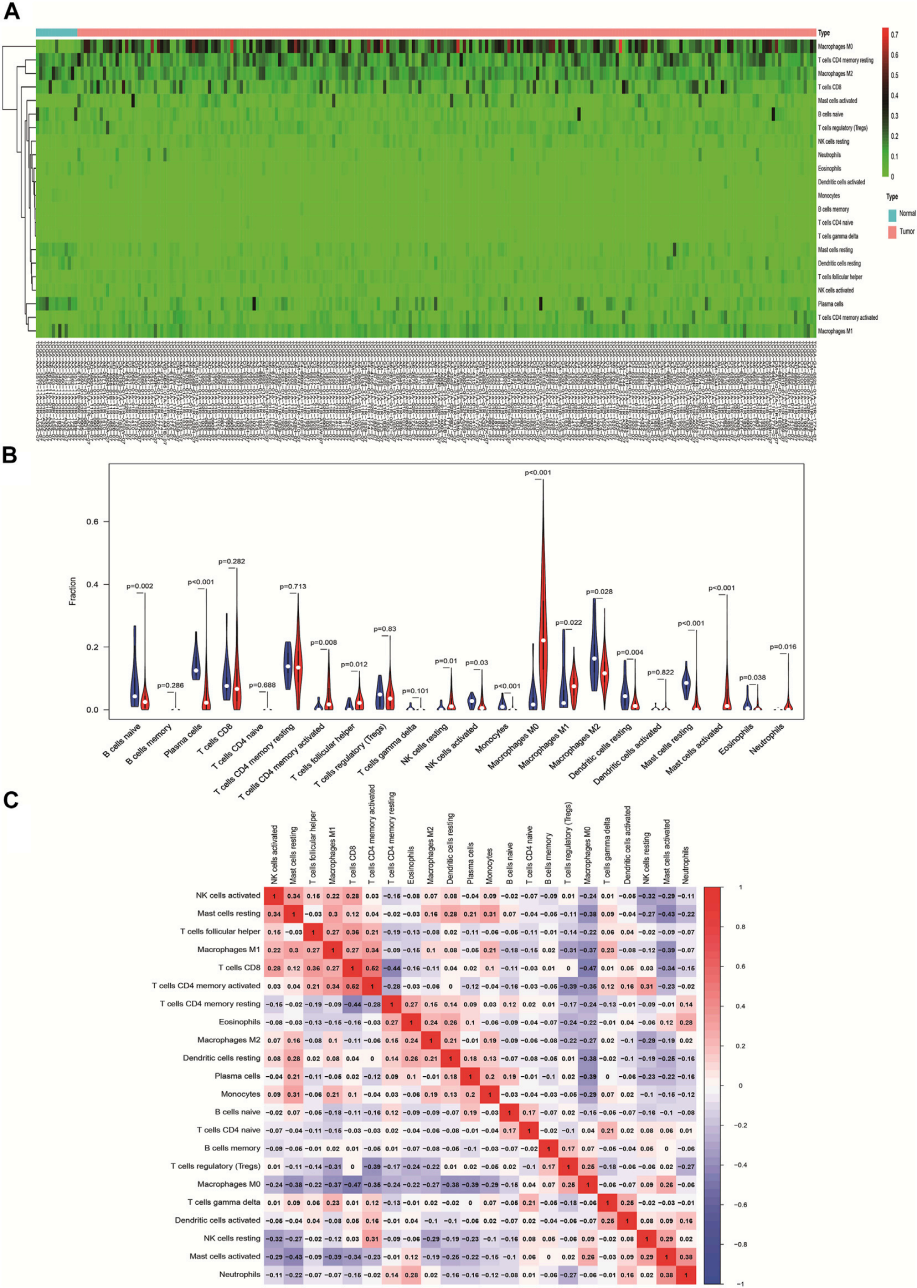
Landscape of immune infiltration in normal and CRC tissues based on TCGA data. **(A)** Heat map of the 22 immune cell expressions. The genes shown in red are upregulated, and the genes in green are downregulated. **(B)** Violin map of the 22 immune cell expressions in normal and CRC tissues. **(C)** Correlation matrix of 22 immune cell expressions based on TCGA data. The redder the color, the higher the positive correlation. The bluer the color, the higher the negative correlation.

We used data of TCGA database to validate the results of immunocyte infiltration of GEO data. The distribution of immune cells in each sample is plotted as a histogram, with which it can be visually found that the distribution of immune cells in different samples is significantly different and consistent with the GEO results (Supplementary Figure S1). Compared with normal tissues, CRC tissues contained a high proportion of M0 macrophages and activated T cells CD4 memory, whereas the M2 macrophages and resting T cells CD4 memory fraction was relatively low in cancer (Figure 3A). From the violin map, we can also see that naive B cells, plasma cells, activated NK cells, monocytes, M2 macrophages, resting dendritic cells, resting mast cells, and eosinophils are expressed higher in normal tissues than in cancer tissues (*p* < 0.05). The activated T cells CD4 memory, T cells follicular helper, resting NK cells, M0 macrophages, M1 macrophages, activated mast cells, and neutrophils are mainly expressed in cancer tissues (*p* < 0.05) (Figure 3B). The correlation heat map results are basically consistent with GEO results (Figure 3C).

### The Prognostic and Clinical Values of Tumor-Infiltrating Immune Cells in CRC

**FIGURE 4:**
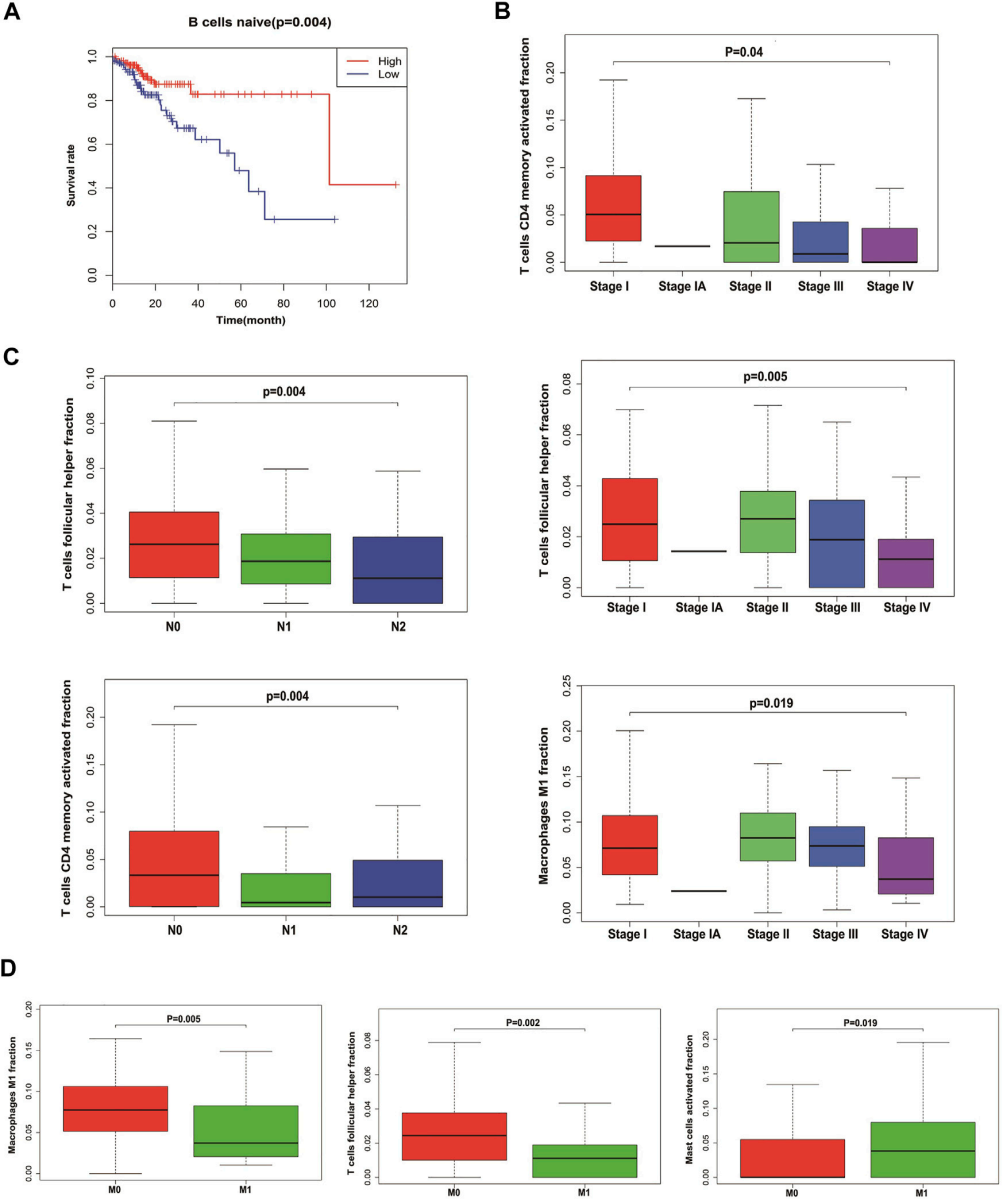
Relationship between tumor-infiltrating immune cells and clinical characters of CRC. **(A)** Survival plots of naive B cells. Data were analyzed using the Kaplan–Meier plotter. Patients with naive B cell expression above the median are indicated in the red line and below the median in the green line. **(B)** Immune cells with the highest correlation with CRC stages. **(C)** Immune cells with the highest correlation with lymph node metastasis in CRC. **(D)** Immune cells with the highest correlation with distant metastasis in CRC.

We analyzed the relationship between infiltrating immune cells and the prognosis of patients with CRC. Through Kaplan–Meier analysis, we found that only naive B cells had statistical significance in the prognosis of patients (Figure 4A). Associated with the tumor stage are activated T cells CD4 memory, T cells follicular helper, and M1 macrophages (Figure 4B). We used Wilcoxon’s test to find that activated T cells CD4 memory and T cells follicular helper showed significant differences in lymph node metastasis and non-metastasis groups (Figure 4C). Also, the cells associated with tumor hematogenous metastasis are T cells follicular helper, M1 macrophages, and activated mast cells (Figure 4D).

### Establishing and Validating a Nomogram Model to Predict CRC Prognosis

**FIGURE 5:**
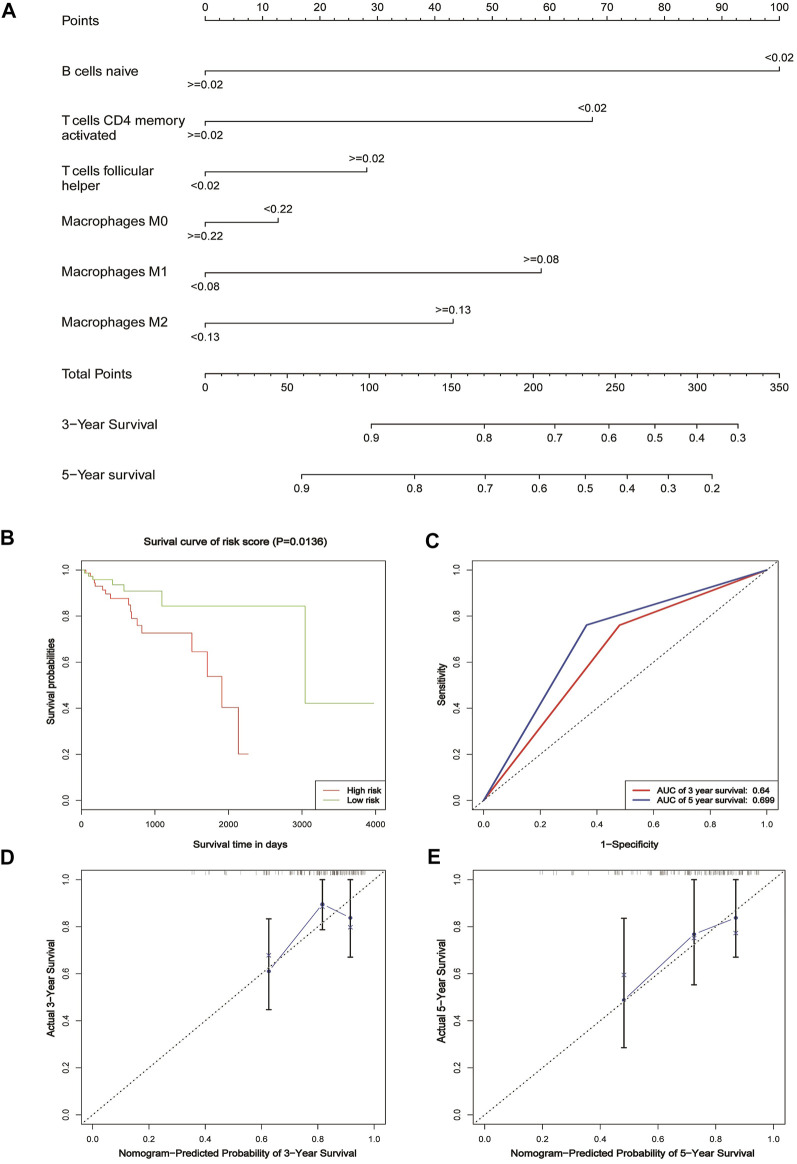
Nomogram for patients with CRC. **(A)** Nomogram for predicting 3- and 5-year survival for CRC patients based on tumor-infiltrating immune cells. **(B)** Kaplan–Meier estimates of patients’ survival status and time using the median risk score cut-off, which divided patients into low-risk and high-risk groups based on the training cohort. **(C)** ROC analysis of the sensitivity and specificity of the survival time by the tumor-infiltrating immune cells based on the risk score for the training cohort. **(D)** Calibration curve for the prediction of 3-year overall survival based on the training cohort. **(E)** Calibration curve for the prediction of 5-year overall survival based on the training cohort.

In order to predict the overall survival rate of CRC patients individually, we selected the independent prognostic factor naive B cells as one variable. The other variables differentially expressed in different clinical traits were screened out by the Wilcox test, as shown in Figure 4. The immune cells with too low expression levels were removed. Finally, we used naive B cells, activated T cells CD4 memory, T cells follicular helper, M0 macrophages, M1 macrophages, and M2 macrophages to establish a nomogram model to predict the overall survival rate of CRC patients for 3–5 years (Figure 5A). In the training group, Kaplan–Meier analysis showed that stratified risk factors can distinguish the overall prognostic survival rate (*p* = 0.0136) (Figure 5B). The AUC values predicting 3-year and 5-year survival rates were 0.64 and 0.699, respectively (Figure 5C). The calibration curve verifies model credibility (Figures 5D,E). In the validation group, Kaplan–Meier analysis showed that stratified risk factors could better distinguish the overall prognostic survival rate (*p* = 0.0026) (Supplementary Figure 2A), and the AUC values predicting 3-year and 5-year survival rates were 0.836 and 0.844, respectively (Supplementary Figure 2B).

### Different Markers Expressed Differently in CRC and Normal Tissues

**FIGURE 6:**
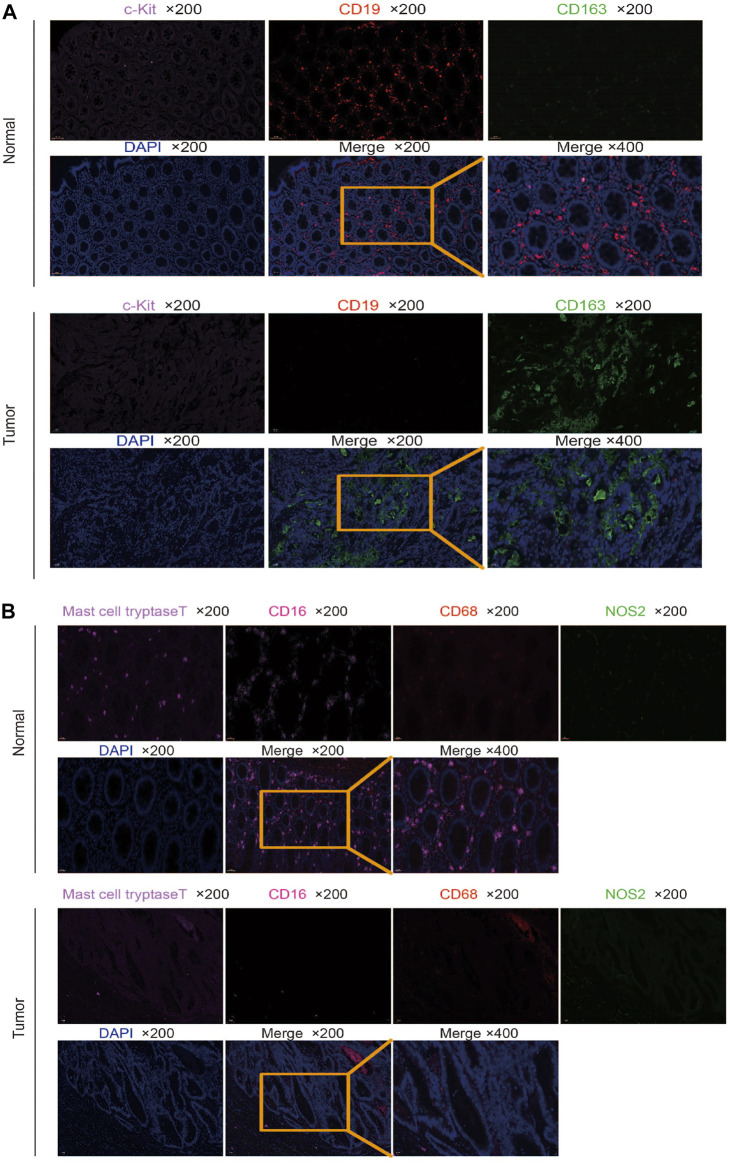
Multiplex immunofluorescence detection of different marker expressions. **(A)** Detection of CD19, CD163, and C-kit expression in tumor and normal tissues by multiplex immunofluorescence. **(B)** Detection of CD68, NOS2, mast cell tryptase, and CD16 expressions in tumor and normal tissues by multiplex immunofluorescence.

We used multiple immunofluorescence techniques to detect the expression of CD19, CD163, C-kit, CD68, NOS2, mast cell tryptase, and CD16 markers in colorectal cancer tissues and surrounding normal tissues on the same slide. C-kit and CD19 were mainly expressed in normal tissues, and their weak expression in tumors was consistent with the results of bioinformatics analysis, while CD163 was more strongly expressed in tumors than in normal tissues (Figure 6A). Mast cell tryptase was widely expressed in tumor parenchyma but weakly and regionally strongly expressed in normal tissues. CD16 expression was stronger in normal tissue than in tumor tissue. The expression of CD68 and NOS2 was stronger in tumor stroma than in normal tissue but weaker in tumor parenchyma than in normal tissue (Figure 6B).

## Discussion

Tumor immunotherapy is an important therapy after surgery, radiotherapy, chemotherapy, and targeted therapy. Immune drug therapy has great challenges and great potential (Ventola, 2017). It is important to evaluate the infiltration of immune cells in different tumors. Based on the gene expression data in TCGA and GEO databases, this study analyzed the distribution of tumor-infiltrating immune cells in CRC. The relationship between infiltrating immune cells and clinical characteristics was also studied to reveal its prognostic value in CRC. Finally, we established a nomogram using infiltrating immune cells to assess the prognosis of patients.

By comparing the GEO datasets and TCGA data results in immune cell infiltration, we found that the highly expressed immune cells in CRC were M0 macrophages, M1 macrophages, activated mast cells, and neutrophils. Naive B cells, M2 macrophages, and resting mast cells are highly expressed in normal tissues. The macrophages can be classified into classical M1 and M2 according to their functions (Orihuela et al., 2016). M1 macrophages participate in the inflammatory response and anti-tumor immunity (Ubil et al., 2018), while M2 macrophages can promote tumor development (Genard et al., 2017). M0 macrophages are formed by mononuclear cells that have not yet to the M1 or M2 macrophage polarization (Zajac et al., 2013). The macrophages in the tumor stroma are called tumor-associated macrophages (TAM) (Petty et al., 2021). Some studies have shown that TAM may have an M2 phenotype, which is closely related to tumor angiogenesis and lymphangiogenesis, and is involved in the process of tumor occurrence, growth, invasion, and metastasis (Riabov et al., 2014). Other studies have shown that TAM has an M1 phenotype and can secrete immunomodulatory factors and enzymes to play an anti-tumor immune role (Hobson-Gutierrez and Carmona-Fontaine, 2018). In breast cancer, TAM plays a dominant role in tumor promotion, and the expression of TAM is negatively correlated with the prognosis of patients (Zhao et al., 2017). However, in lung cancer, the results were just the opposite, i.e., TAM infiltration was positively correlated with the prognosis of patients (Conway et al., 2016). The role of TAM in tumors is not clear. The results of this study suggested that the expression of M2 macrophages in CRC tissues was lower than that in normal tissues, but it was not related to TNM stage, tumor size, and presence or absence of hematological and lymphatic metastasis. The expression of M1 macrophages in the tumor was higher than that in normal tissue and correlated with lymphatic metastasis. This result is consistent with another report using the CIBERSORT method to analyze immune cell infiltration in CRC tissues (Xiong et al., 2018). These results suggest that M1 macrophages and M2 may play different roles in different tumors or at different stages of tumors. No matter which research results show the important role of macrophages in tumors, further experiments are needed to verify their function.

As seen from the correlation heat map, M2 macrophages are significantly negatively correlated with activated mast cells and positively correlated with resting mast cells, and resting mast cells and activated mast cells are negatively correlated. Many literature studies have reported that mast cells have the effect of promoting cancer (Molderings et al., 2017; Aponte-Lopez et al., 2018; Komi and Redegeld, 2020), and we speculate that there may be a regulatory relationship among them, which plays a role in the tumor together.

Kaplan–Meier analysis showed that only naive B cells had statistical significance in the prognosis of patients. A naive B cell is a B cell that has not been exposed to an antigen. Once exposed to an antigen, the naive B cell either becomes a memory B cell or a plasma cell that secretes antibodies specific to the antigen that was originally bound. Little has been reported about the relationship between the initial B cells and the tumor, and there may be unknown functions awaiting further exploration.

The occurrence and development of CRC have great complexity and heterogeneity in cell origin, histological grade, clinical stage, recurrence, and metastasis. There is still a lack of practical, inexpensive prognostic assessment tools in clinical work. The nomogram is a mapping method based on the results of multivariate analysis. It can integrate multiple clinical–pathological indicators to determine the probability of a certain clinical event in a particular individual (Balachandran et al., 2015). It has become a relatively simple and accurate prediction method and has been applied to the prognosis of tumors. We used these six risk factors, naive B cells, activated T cells CD4 memory, T cells follicular helper, M0 macrophages, M1 macrophages, and M2 macrophages, to construct a nomogram to assess the patient’s 3-year and 5-year survival rate. The nomogram model established in this study divided the patients with CRC into a high-risk group and low-risk group, which can better distinguish the prognosis survival rate of patients. In the verification group, the model AUC value reached 0.844, and the correction curve also showed that the model had a good consistency with the actual incidence. The nomogram model constructed by this study can predict the prognosis of individual patients and individualized treatment for patients with CRC.

We verified marker expression with multiplex immunofluorescence. It was found that naive B cells and resting mast cells were mainly found in normal tissues, but few in tumor tissues, which was consistent with the results of bioinformatics analysis. However, some results are inconsistent with bioinformatics analysis. The M2 macrophage cells were mainly found in tumor tissues. The activated mast cells were widely infiltrated, but weakly in tumor parenchyma, and have a strong regional expression in normal tissues. The neutrophils were mainly found in normal tissues compared to tumor tissues. The M0 macrophage and M1 macrophage cells in tumor stroma were stronger than that in normal tissue, and the infiltration in tumor parenchyma was weaker than that in normal tissue. The inconsistency between the multiple immunofluorescence verification results and the bioinformatics analysis results may be because the data in TCGA and GEO databases are RNA expression, and RNA expression may be inconsistent with protein expression. In addition, the expression of different markers in tumor parenchyma and stroma is different. Bioinformatics analysis is for the whole tissue, while multiple immunofluorescence can see the location and number of different immune cells in different regions. Moreover, different patients have different conditions, such as the impact of radiotherapy and chemotherapy, which may also lead to the existence of inconsistency.

## Data Availability

The original contributions presented in the study are included in the article/Supplementary Material, further inquiries can be directed to the corresponding authors.
