# Supplementary figures and images for: The Landscape of the Tumor-Infiltrating Immune Cell and Prognostic Nomogram in Colorectal Cancer
Source: Front Genet. 2022 May 12;13:891270. doi: 10.3389/fgene.2022.891270 (PMC9133796; doi:10.3389/fgene.2022.891270)

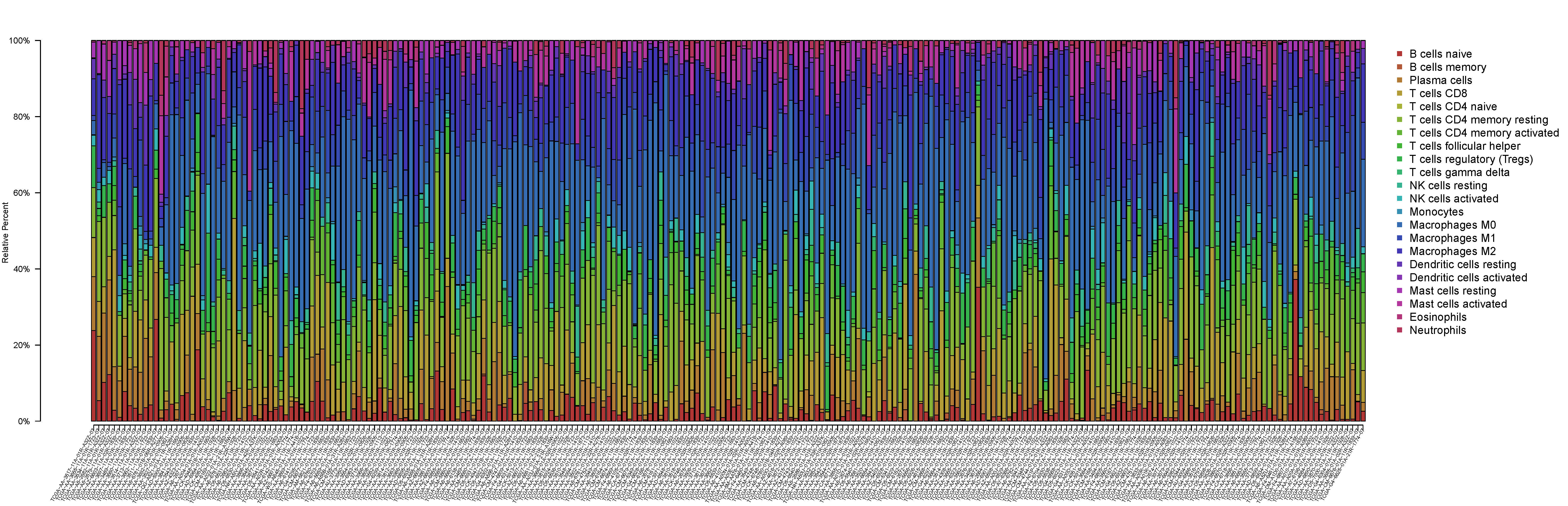

Supplement: Supplementary file 1 [file Image1.TIFF]

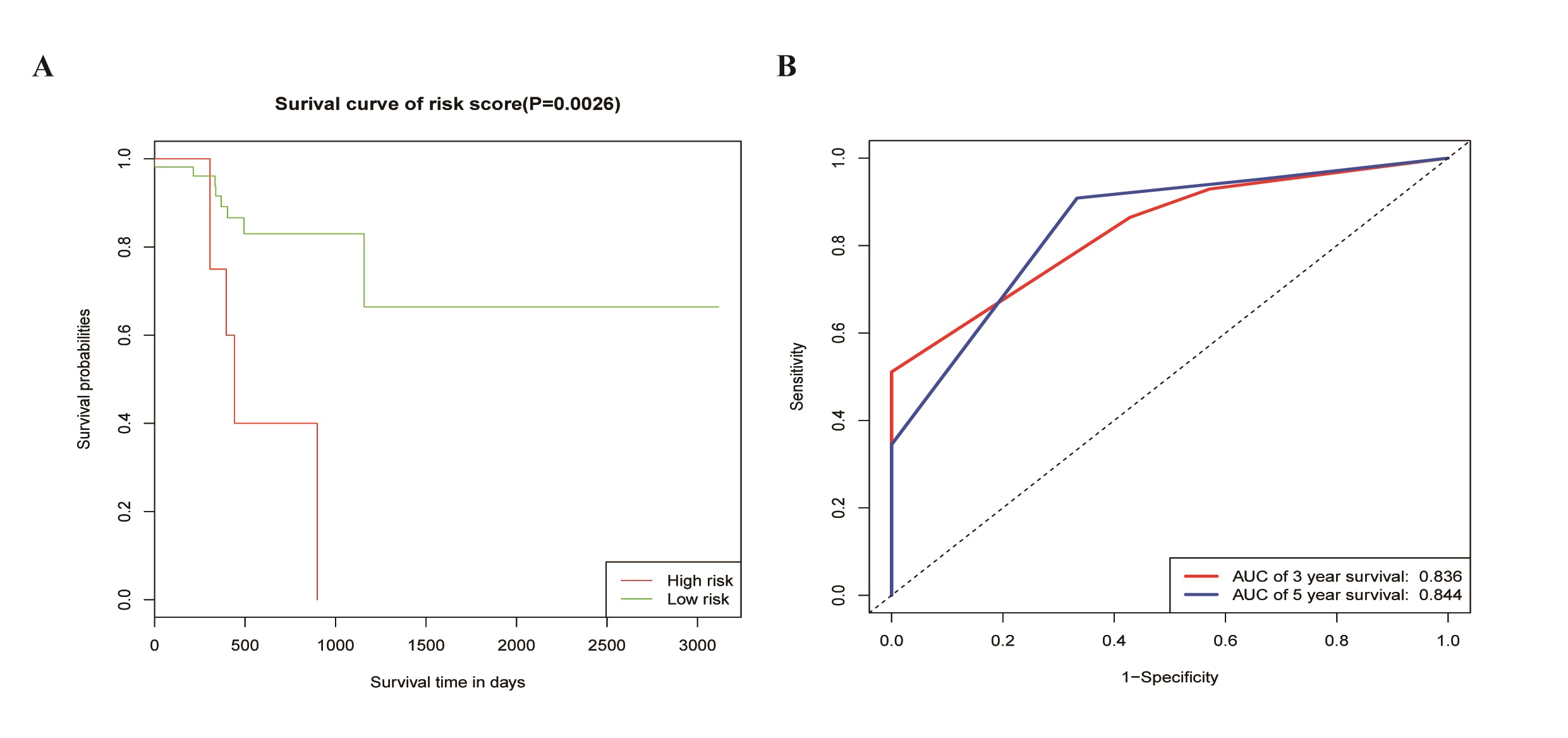

Supplement: Supplementary file 2 [file Image2.JPEG]
