# Supplementary material for: The Landscape of the Tumor-Infiltrating Immune Cell and Prognostic Nomogram in Colorectal Cancer
Source: Front Genet. 2022 May 12;13:891270. doi: 10.3389/fgene.2022.891270 (PMC9133796; doi:10.3389/fgene.2022.891270)
Supplement: Supplementary file 4 [file DataSheet1.docx]

**Supplementary FIGURE 1** The composition of immune infiltration in normal and CRC tissue based on TCGA data. A bar represents the immune cells distributed within a sample.

**Supplementary FIGURE 2** Nomogram construction for patients with CRC. **(A)** Kaplan-Meier estimates of patients' survival status and time using the median risk score cut-off which divided patients into low-risk and high-risk group based on training cohort. **(B)** Receiver operating characteristic (ROC) analysis of the sensitivity and specificity of the survival time by the tumor-infiltrating immune cells based on risk score for training cohort. **(C)** The calibration curve for the prediction of 3-year overall suvival based on training cohort. **(D)** The calibration curve for the prediction of 5-year overall suvival based on training cohort.
